# Fast simulation of hemodynamics in intracranial aneurysms for clinical use

**DOI:** 10.1007/s00701-025-06469-9

**Published:** 2025-03-03

**Authors:** Daniel Deuter, Amer Haj, Alexander Brawanski, Lars Krenkel, Nils-Ole Schmidt, Christian Doenitz

**Affiliations:** 1 Klinik und Poliklinik für Neurochirurgie, University Hospital Regensburg, Franz-Josef-Strauß-Allee 11, 93053 Regensburg, Germany; 2 Regensburg Center of Biomedical Engineering (RCBE), OTH Regensburg and University of Regensburg, 93053 Regensburg, Germany

**Keywords:** CFD, Intracranial aneurysm, Intraoperative, Rupture risk evaluation, Thin-walled regions, Local risk assessment

## Abstract

**Background:**

A widely accepted tool to assess hemodynamics, one of the most important factors in aneurysm pathophysiology, is Computational Fluid Dynamics (CFD). As current workflows are still time consuming and difficult to operate, CFD is not yet a standard tool in the clinical setting. There it could provide valuable information on aneurysm treatment, especially regarding local risks of rupture, which might help to optimize the individualized strategy of neurosurgical dissection during microsurgical aneurysm clipping.

**Method:**

We established and validated a semi-automated workflow using 3D rotational angiographies of 24 intracranial aneurysms from patients having received aneurysm treatment at our centre. Reconstruction of vessel geometry and generation of volume meshes was performed using AMIRA 6.2.0 and ICEM 17.1. For solving ANSYS CFX was used. For validational checks, tests regarding the volumetric impact of smoothing operations, the impact of mesh sizes on the results (grid convergence), geometric mesh quality and time tests for the time needed to perform the workflow were conducted in subgroups.

**Results:**

Most of the steps of the workflow were performed directly on the 3D images requiring no programming experience. The workflow led to final CFD results in a mean time of 22 min 51.4 s (95%-CI 20 min 51.562 s–24 min 51.238 s, *n* = 5). Volume of the geometries after pre-processing was in mean 4.46% higher than before in the analysed subgroup (95%-CI 3.43–5.50%). Regarding mesh sizes, mean relative aberrations of 2.30% (95%-CI 1.51–3.09%) were found for surface meshes and between 1.40% (95%-CI 1.07–1.72%) and 2.61% (95%-CI 1.93–3.29%) for volume meshes. Acceptable geometric mesh quality of volume meshes was found.

**Conclusions:**

We developed a semi-automated workflow for aneurysm CFD to benefit from hemodynamic data in the clinical setting. The ease of handling opens the workflow to clinicians untrained in programming. As previous studies have found that the distribution of hemodynamic parameters correlates with thin-walled aneurysm areas susceptible to rupture, these data might be beneficial for the operating neurosurgeon during aneurysm surgery, even in acute cases.

## Introduction

Intracranial aneurysms occur with a prevalence of about 3% [23, 73, 79]. Whilst many aneurysms remain stable in a state of equilibrium [9, 67, 81], the risk of rupture with following subarachnoid hemorrhage (SAH) weighs heavily due to high morbidity and mortality [31, 32] with median mortality rates of up to 44% in Europe [53].

Hemodynamics is broadly accepted as one of the key factors in the formation, growth and rupture of aneurysms [20, 64, 66, 72, 75]. Therefore, multiple authors investigated the correlation between aneurysm hemodynamics and rupture [12, 13, 46, 55, 85]. Systematic meta-analyses found correlations between aneurysm rupture and low Wall Shear Stress (WSS) [1, 28, 89], the shear stress acting tangential to the endothelial cell surface based on the frictional forces of blood [43, 63]. Pathological WSS values are thought to provoke endothelial dysfunction resulting in various effects like flow-driven inflammation and biochemical alterations within the endothelial layer [62, 75, 80].

**Fig. 1:**
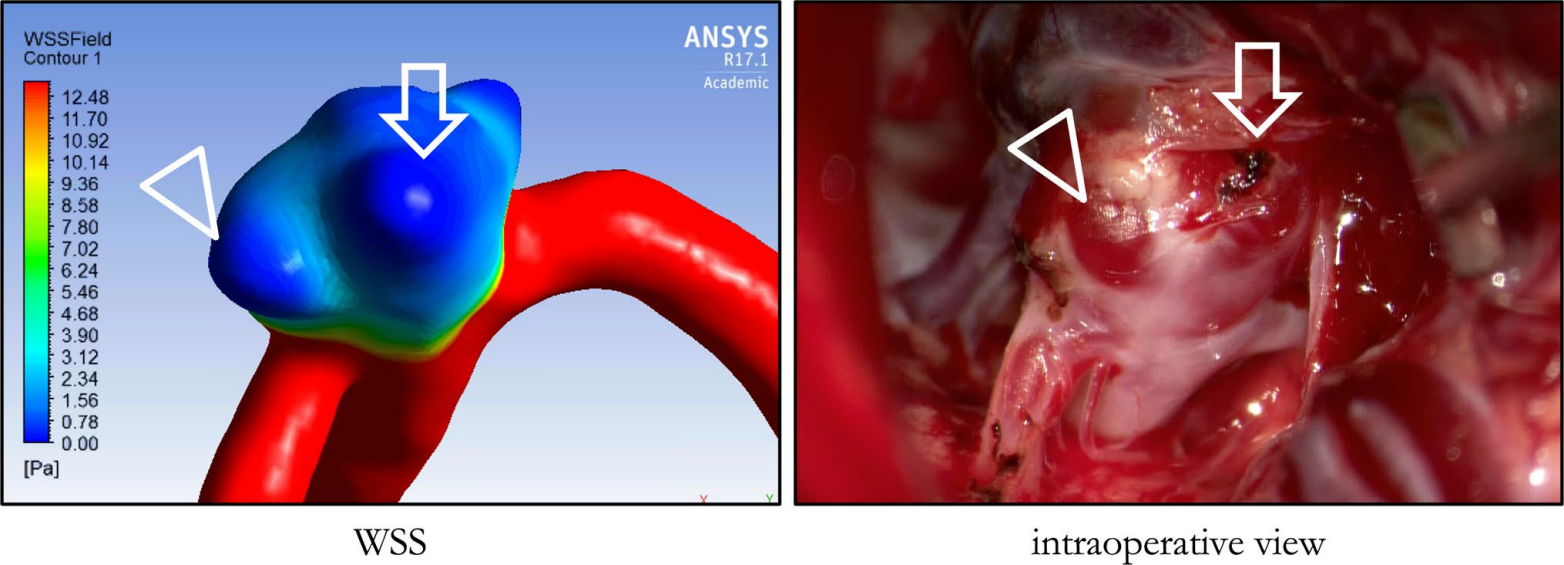
Case of a 39 years old patient with a right-sided M1 bifurcation aneurysm: CFD simulation of the aneurysm (left) compared to the intraoperative view during aneurysm surgery (right). This 39 years old patient received Magnetic Resonance Imaging (MRI) due to headaches of unclear etiology incidentally revealing a right-sided M1 aneurysm. Ancillary, the patient suffered from terminal renal insufficiency due to Granulomatosis with Polyangiitis (GPA) requiring intermittent hemodialysis. After diagnostic angiography, indication was set for surgical clipping of the aneurysm which was planned only several days after the angiography. Preoperatively, CFD simulations were performed to make the operating neurosurgeon benefit from hemodynamic data directly in the OR. The left image shows the WSS map obtained from CFD simulations, the right image the intraoperative view through the operating microscope. Arrow and triangle mark two blebs with locally lowered WSS-values which corresponded well with intraoperatively thin-walled areas appearing translucent under the operating microscope. Export to the neuronavigation system was feasible, but not used in this case

Apart from aneurysm-wide rupture risk assessment, another promising application of hemodynamic simulations is the detection of vulnerable thin areas of the aneurysm wall prone to rupture [18, 25, 37, 40, 41, 44, 70, 71] (see Fig. 1). Correlations between thin-walled areas, defined as intraoperative translucent regions of aneurysms and low WSS-rates have been found [18, 41] as well as correlations between low WSS and local rupture points [70]. Associations between ruptured thin-walled aneurysm blebs and low WSS as well as high Oscillatory Shear Index (OSI) were found compared to non-ruptured blebs [44].

One major tool to study hemodynamic forces is Computational Fluid Dynamics (CFD). Incompressive flow, physically described by the Navier–Stokes-equations, can be simulated by solving these equations with software applications numerically for each point of a geometry represented by a volume mesh [19, 47, 52, 74]. As CFD simulations are still time consuming and require highly qualified experts, the method often remains restricted to computational laboratories, which is one main reason that prevents CFD to come into clinics. There it could provide valuable information for planning of aneurysm surgery and intraoperative dissection strategy, especially with regard to aneurysm areas that will not be visible during surgical exposure due to anatomic reasons.

The aim of this study was to develop a fast and intuitive workflow for clinical routine, which leads to robust results in less than half an hour to make clinicians benefit from hemodynamic simulations even in acute cases. An exemplary case on how CFD might be incorporated in the clinical setting is described in the legend of Fig. 1.

## Methods and materials

This study was approved by the local ethics committee of the University Hospital of Regensburg (protocol code 24–3666-104) and was performed in accordance with the declaration of Helsinki and its further amendments.

We established a semi-automated, batch-script-based workflow using AMIRA 6.2.0 (FEI Visualization Sciences, France), ICEM 17.1 (ANSYS Inc., USA) and ANSYS CFX 17.1 (ANSYS Inc., USA). For the development of the workflow and its validation, we retrospectively used three-dimensional rotational angiograms (3DRA) of 24 MCA aneurysms from 23 patients having undergone surgical or endovascular aneurysm treatment at our center with an isotropic voxel size of 0.216 mm. 13 patients (57%) presented with subarachnoid hemorrhage, but not all from the specific aneurysm investigated in this study. Calculations were performed using a commercial standard machine (Intel Core i3 1.8 GHz, 8 GB RAM, NVIDIA GeForce GT 740 M).

The workflow to perform CFD simulations consists of various steps, mainly 1.) geometric reconstruction of the vessel anatomy leading to volume meshes representing the vessel geometry, 2.) definition of boundary conditions for inlets and outlets and setup of physics, 3.) flow simulation in the proper sense and 4.) post-processing and visualization of the obtained results. As the Navier Stokes equations can’t be mathematically exactly solved for the reconstructed patient specific geometry in a whole, the vessel geometry has to be divided into volume meshes/ grids consisting of numerous elements. The CFD software package (“solver”) approximates the solutions for each element or volume iteratively until a given convergence is obtained [19].

**Fig. 2:**
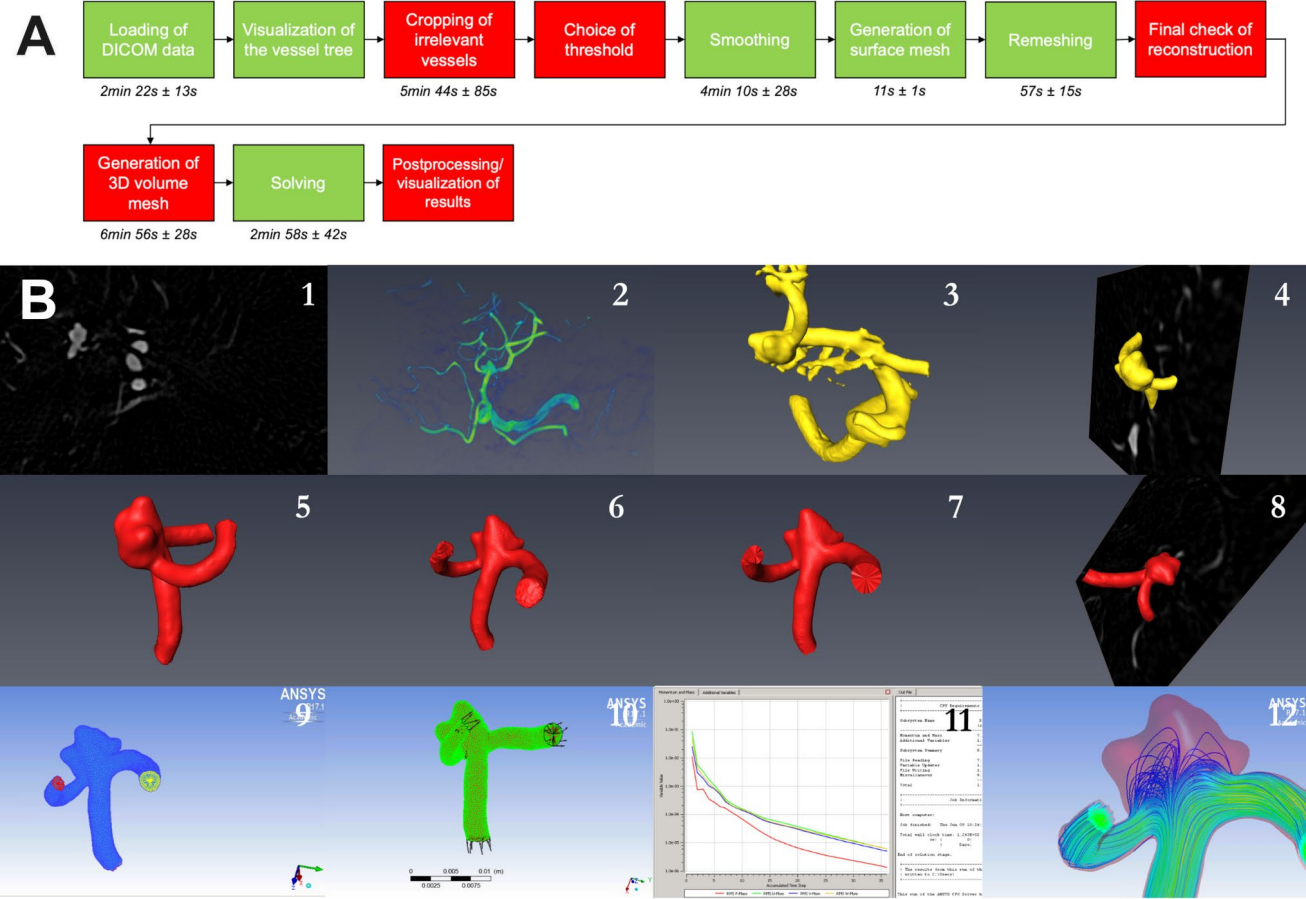
Overview over the workflow (Fig. 2A) and single steps shown for an exemplarily aneurysm of the M1 bifurcation (Fig. 2B): **A**) The indicated time represents the mean time ± standard deviation needed to perform a specific step as obtained from later time tests from five aneurysms (please see also “4. Time requirements” in the methods- and results section). These aneurysms were processed de novo to avoid learning effects in the individual geometry. Automatized steps are shown in red, manual steps in green. **B**) Individual steps shown for an exemplarily aneurysm of the M1 bifurcation. 1 Loading of DICOM dataset (automatized); 2 first visualization of the vessel geometry (automatized); 3 cropping of irrelevant vessels (manual); 4 choice of threshold in direct overlay with the 3DRA dataset (manual); 5 smoothing (automatized); 6 generation of the surface mesh (automatized); 7 smoothing of the surface mesh and remeshing (automatized); 8 final check of the reconstruction (manual); 9 generation of the 3D volume mesh (manual/ semi-automated); 10 setup of physics (manual); 11 solving (automatized); 12 postprocessing and visualization of the results (manual)

The detailed steps of our CFD workflow are shown in Table 1 and Fig. 2A as well as the used software packages (Table 1). Figure 2B shows the specific steps of the workflow in an exemplarily case. In the following, these steps are described in more detail.

**Table 1:** Steps of the CFD workflow in detail: For geometric reconstruction, AMIRA was used, for calculation of volume meshes ICEM. Flow simulations were performed using ANSYS CFX. General steps are shown in the first column, the second column shows the specific steps in more detail. The degree of automatization is shown in the last column

General steps	Steps in detail	Degree of automatization
1. Geometric reconstruction of the vessel geometry (AMIRA)	1: Loading of DICOM dataset	automatized
	2: 3D-Visualization of the vessel geometry	automatized
	3: Cropping of unnecessary vessels leading to the vessel geometry only focusing on the relevant vessels	manual
	4: Check of the threshold used for binarization of the vessel geometry	manual
	5: Smoothing of the dataset	automatized
	6: Generation of a surface mesh	automatized
	7: Smoothing of the surface mesh and remeshing leading to surface meshes with very regular triangles optimal for further CFD simulations	automatized
	8: Check of final reconstruction	manual
2. Volume meshing (ICEM)	9: Calculation of 3D-volume meshes based on the surface meshes	manual/ semi-automated
3. Flow simulation (ANSYS CFX)	10: Setup of physics at the inlets and outlets and boundary conditions	manual
	11: Solver (iterative calculation of the Navier–Stokes-equations for each point of the geometry)	automatized
	12: Post-processing and visualization of results	manual

### Geometric reconstruction of the vessel geometry

 In this study, only 3DRAs were used, but not Computed Tomography Angiographies (CTA) or Magnetic Resonance Angiographies (MRA). As 3DRA is accepted to be the gold-standard regarding the adequate depiction of the vascular and aneurysm geometry, this ensures correct geometric reconstruction urgently needed for reliable CFD results. Nevertheless, the usage of CTA- or MRA images would be generally also possible with the presented workflow after further pre-processing, even if this was not part of this study.

After loading of the 3DRA DICOM files into the program, a first overview over the patient’s vessel geometry was obtained using a volume-rendering module. A Region of Interest (ROI) was interactively defined by a 3D box evoking a cropped dataset focusing on this region which leads to a considerable reduction of computational burden and –time for the following steps. For generation of isosurfaces, a threshold for binarization was evaluated in direct overlay with the original 3DRA DICOM files. A Sobel filter was attached to the raw dataset, as gradient edge detection has been found to guarantee better volume reproducibility than simple thresholding [8]. Artifacts, vessel intersections and “kissing vessels” were removed in direct interaction with the 3D-visualization by using a free-hand lasso tool. Special attention should be paid at an adequate reconstruction of the vessel geometry as this is crucial to obtain reliable CFD results and can be time-consuming or problematic due to “kissing vessels” or imaging artifacts. Parent vessels were cropped at the beginning of the M1 segment, outlets at a length of a minimum of 5 vessel diameters. Subsequently, a 3D-Gauss-Cernel was applied with a standard deviation of 0.4 and a non-local-means filter [10]. More aggressive smoothing was avoided due to the risk of volumetric shrinking. After smoothing, a second and final threshold was evaluated in overlay with the raw datasets of 3DRAs to compensate even moderate shrinking artifacts. Tetrahedral surfaces were generated and smoothed with moderate values (one iteration, lambda 0.3). Remeshing was performed using an algorithm by Zilske et al., implemented in AMIRA [91] leading to regular and uniform triangles of the surface mesh. Inlets and outlets were cut to plane section by removing surface tetrahedrons and filling the newly formed holes. To guarantee realistic geometric results, final surface models were compared with original 3DRA in direct overlay. The whole work pipeline in AMIRA was semi-automatized by creation of a.tcl script.

### Volume meshing

 Surfaces were transferred to ICEM using the.stl format. After subdivision of inlets and outlets, volume meshes were created using a robust octree approach protecting the existing parts of the surface mesh. At the inner wall, three prism layers growing with exponential law were created with a height ratio of 1.2 and a initial height of 0.02 mm. The usage of prism layers leads to a good compromise between computational time for solving and a good resolution of flow phenomena near the vessel border where slower velocities can be found due to the parabolic blood flow profile. The WSS, one main parameter at interest in this study, is the tangential force on the endothelium arising from the frictional forces between the blood and the vessel wall as well as in the blood fluid itself [63].

### Flow simulation

 The simulation was modeled with rigid walls with non-slip condition. Constant inlet flow rates were defined with static outlet pressures of 0 Pa. In general, inlet flows can be defined based on patient-specific values obtained from for example Transcranial Doppler Ultrasound (TCD) or on standard parameters from literature (see for instance [22, 54, 69, 88]). To mimic the intraoperative situation during aneurysm clipping, in this study, an inflow velocity of 28 cm/s was defined as general anesthesia was found to relevantly reduce MCA flow velocity [26, 48]. For definition of outlet pressures, flow distribution or coupling of the simulation to a 1D-model of the vascular tree might provide better options than a zero-pressure condition [7]. Blood was modeled as a non-Newtonian fluid, following a power-law-model with dynamic viscosity [15, 38, 39]. As convergence criteria, a residual target of 0.00001 was defined. Solving was performed using ANSYS CFX. We performed steady-state simulations of streamlines, pressure, WSS and Wall Shear Gradient (WSSG). Post-processing was performed using CFX Post leading to color-coded streamlines and surface plots. Export of the results to the intraoperative neurosurgical navigation software (Brainlab, Munich, Germany) was established.

Subsequently, this setting was tested for stability and accuracy using the following setup, as shown in Table 2. Because correlations between low WSS and thin-walled regions could be found consistently in steady state as well as pulsatile simulations [41], only steady state simulations were investigated.

**Table 2:** Setup used for the validation of the workflow: We examined the influence of smoothing operations, surface and volume mesh sizes, geometric mesh quality and the time needed for CFD simulations using the presented workflow

General steps for validation	Steps in detail
1. Evaluation of the volumetric impact of smoothing operations	
2. Evaluation of the impact of the mesh size on CFD results (grid convergence)	a) Surface grid convergence
	b) 3D volume mesh grid convergence based on the medium surface mesh
	c) 3D volume mesh grid convergence based on the finest surface mesh
3. Geometric mesh quality	
4. Time requirements	

### Volumetric impact of smoothing operations

To exclude geometric artifacts due to smoothing and second thresholding, we compared surface contours of the first reconstruction with the processed surface on 10 axial slices, equally distributed over the aneurysm geometry in a subcohort consisting of 5 randomly picked aneurysms. Comparisons of the volumes of the surfaces before and after these operations were performed.

### Impact of mesh size on the CFD results (grid convergence)

 Grid convergence studies are a major tool to assess quality of CFD simulations, especially for the estimation of errors [59, 60] and have also been successfully used in aneurysm CFD [29]. As a mesh with an infinite number of elements leading to an element size of zero, would ideally provide a perfect representation of the given problem, the smaller the number of elements is in the single CFD simulation, the bigger the error will be. Therefore, grid refinement should make the approximated solution more similar to the perfect solution as related errors should asymptotically converge zero [93, 94]. The point when mesh density doesn’t have a big influence on the solution any longer should be found for mesh-independent solutions [19].

**Fig. 3:**
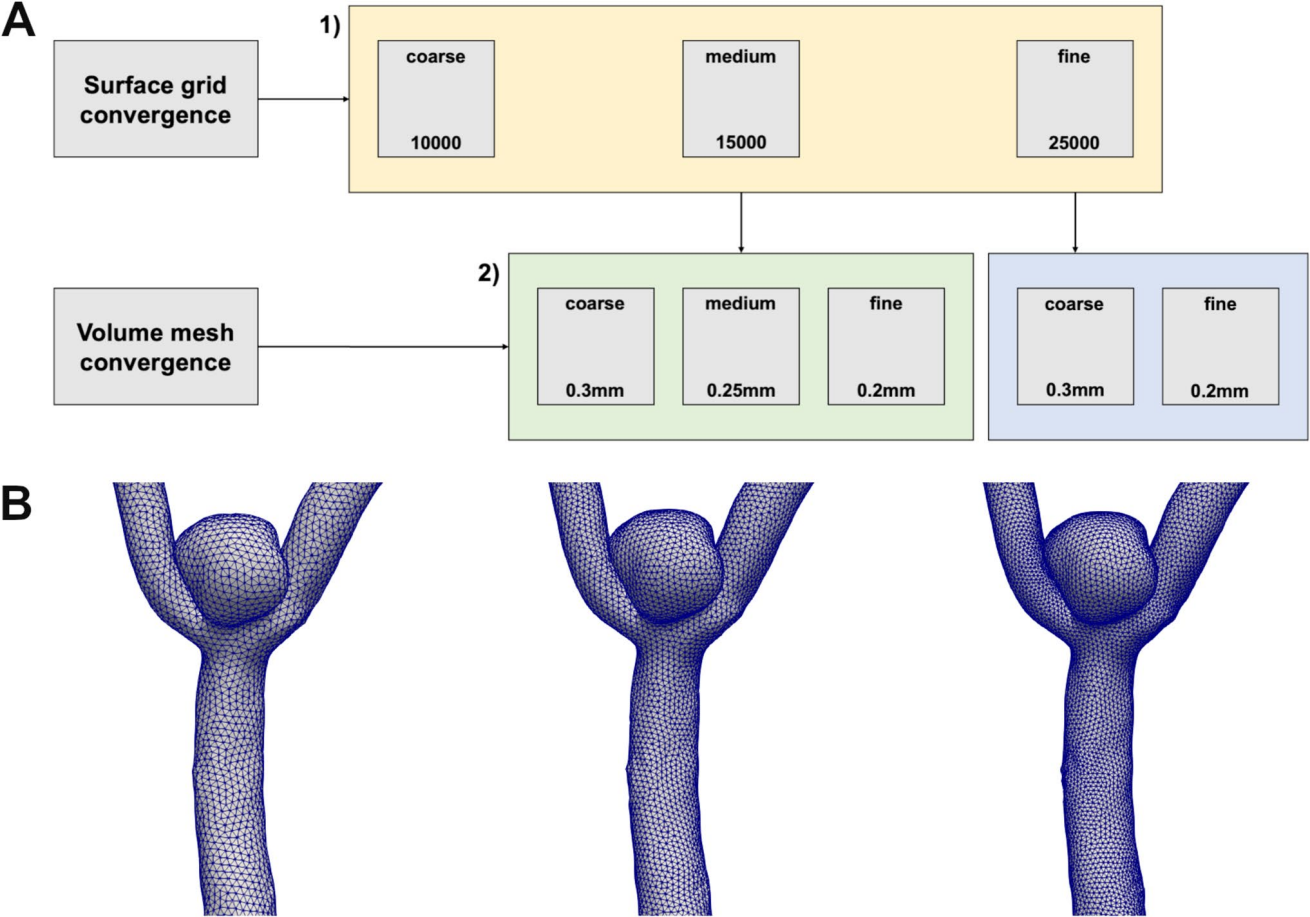
Summary of the methodological workaround used for the assessment of mesh convergence: **A**) Schematic representation of the concept used for the assessment of surface and volume mesh convergence as well as the analyzed meshes. Regarding surface mesh convergence, we defined coarse, medium and fine meshes with the shown element numbers (yellow box). Final meshes used for CFD simulations showed slightly lower numbers of surface elements due to further processing steps. Regarding volume mesh convergence, the volume meshes were defined with the given seed sizes based on the medium surface mesh, initially defined with 15000 elements (green box), and the fine surface mesh, initially defined with 25000 elements (blue box). For volume mesh convergence, all results were analyzed in relation to the finest meshes of this study (seed size 0.2mm based on the surface mesh defined with 25000 elements). **B**)Surface meshes with increasing spatial resolution of an exemplarily M1 bifurcation aneurysm are shown as used for the assessment of mesh convergence (left: coarse surface mesh defined with 10000 surface elements, middle: medium surface mesh defined with 15000 surface elements, right: fine surface mesh defined with 25000 surface elements)

- **Surface mesh convergence:** To evaluate optimal surface mesh sizes, surface meshes were simulated with different numbers of tetrahedrons and identical boundary conditions in a subgroup of 7 aneurysms. The number of tetrahedrons is primarily specified in AMIRA by remeshing and was defined within this step as coarse with 10,000, medium with 15,000 and fine meshes with 25,000 elements. Final meshes showed slightly lower numbers of tetrahedrons due to further processing steps. Seed size for volume meshing was constantly defined as 0.3 mm. We measured local WSS values in 5 defined points on each aneurysm (aneurysm dome vertex, front side, backside, upside area and inlet vessel). For each point, the relative aberrations of the results of the medium and coarse mesh were calculated in relation to the finest mesh. Additionally, the Grid Convergence Index (GCI), originally proposed by Roache [59, 60] and ignoring the absolute difference of elements between the meshes as these heavily influence the results of grid convergence studies, was calculated.
- **Volume mesh convergence based on the medium surface mesh:** Following the same procedure, three different volume meshes derived from the surface mesh initially defined with 15,000 elements were simulated. The coarse volume mesh was defined by a global element seed size of 0.3 mm, the medium mesh with a seed size of 0.25 mm and the fine mesh with a seed size of 0.2 mm. For each mesh, the relative aberration of the results was calculated for each of the above defined points in relation to the finest mesh of the study (25,000 surface elements, seed size 0.2 mm).
- **Volume mesh convergence based on the fine surface mesh:** Finally, two different volume meshes were simulated based on the surface mesh consisting of 25,000 elements. The coarse mesh was defined with a seed size of 0.3 mm, the fine mesh with a seed size of 0.2 mm. For each mesh, the relative aberration of the results was calculated for each of the above defined points in relation to the finest mesh of the study (25,000 surface elements, seed size 0.2 mm). The methodological workaround used for the assessment of mesh convergence is summarized in Fig. 3.

### Geometric mesh quality

 To gain stable results in CFD simulations, geometric elements should offer high regularity. Therefore, equilateral tetrahedrons are optimal elements. The parameter “ICEM quality” implemented in ICEM, gives a quick overview over the geometric mesh quality with values ranging between 0 (very skew elements) and 1 (equilateral elements). Minimum, mean and maximum ICEM mesh quality was exported for the meshes of 20 aneurysms (15,000 surface elements, seed size 0.25 mm).

### Time requirements

 To evaluate the time needed for static calculations, time tests were performed for simulations of 5 aneurysms that were not processed during the preceding steps to avoid learning effects in individual vessel geometries due to repetitive reconstruction (15,000 surface elements, seed size 0.25 mm). We measured the time needed for the whole workflow from loading of the DICOM datasets towards the final postprocessed colormaps when performed by an experienced operator (DD, professional background when performing calculations: Medical student). Inter-performer variability between different operators was not assessed.

## Results

### Volumetric impact of smoothing operations

 In the analyzed subgroup, the mean volume of surface meshes was 482.91mm^3^ (95%-CI 215.21–750.60mm^3^) before smoothing and second thresholding, and 502.34mm^3^ (95%-CI 227.25–777.42mm^3^) afterwards. This represents a mean relative difference of 4.46% (95%-CI 3.43–5.50%) with an increase in volume due to the usage of the second threshold after smoothing operations. This second threshold was used as smoothing operations tend to cause shrinking of the geometry. Visual inspection of borders of obtained surface meshes on axial slices showed good anatomic reproducibility.

### Impact of mesh size on the CFD results (grid convergence)

**Fig. 4:**
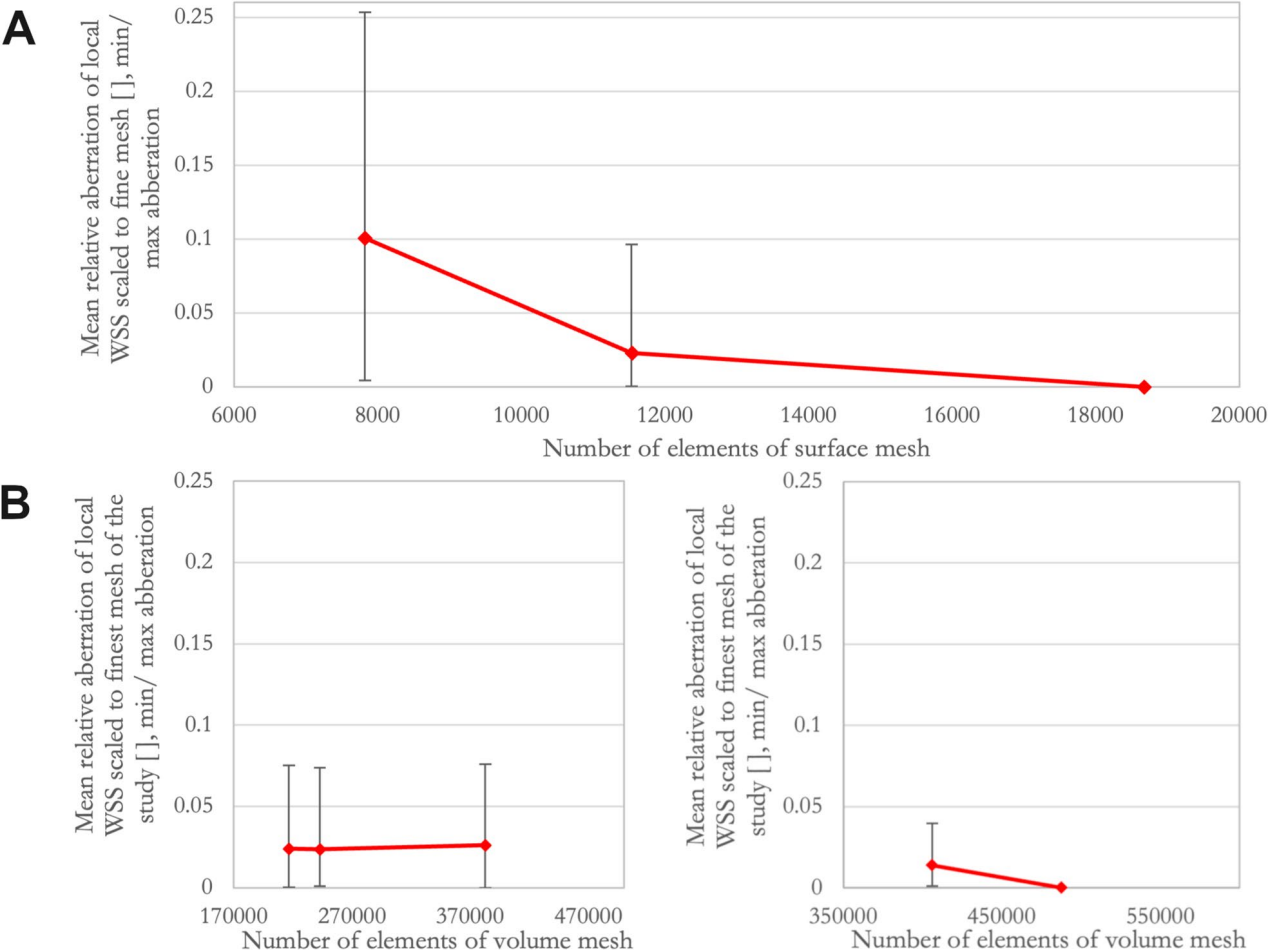
Results from grid convergence: **A**) Results from surface mesh convergence are shown. The diagram indicates the mean relative aberrations of local WSS values scaled to the fine surface mesh. Error bars indicate minimum and maximum aberrations. **B**) Results from volume mesh convergence (left: Results based on the surface mesh defined with 15,000 surface elements; right: Results based on the surface mesh consisting of 25,000 surface elements). The left diagram indicates the mean relative aberrations of local WSS values scaled to the finest mesh of the study (25,000 surface elements, seed size 0.2 mm). Error bars indicate minimum and maximum aberrations. The right diagram indicates the mean relative aberrations of local WSS values scaled to the finest mesh of the study (seed size 0.2 mm). Error bars indicate minimum and maximum aberrations. Numeric values as well as 95%-CIs are also given in Table 3

- **Surface mesh convergence:** In the analyzed subgroup, the mean relative aberration of the measured local WSS values of the medium surface meshes was 2.30% (95%-CI 1.51–3.09%) with a maximum aberration of 9.65% as shown in Fig. 4A. Maximum relative aberrations were found at dome regions with marked low WSS-values. The GCI for the fine mesh was found to be 1.2%.
- **Volume mesh convergence based on the medium surface mesh:** Based on the surface mesh consisting of 15,000 surface elements, mean relative aberration of local WSS values was 2.38% (95%-CI 1.68–3.07%) for the medium meshes with a maximum aberration of 7.39%; mean aberration of local WSS values for the fine meshes was 2.61% (95%-CI 1.93–3.29%) with a maximum aberration of 7.59%. Results are shown in Fig. 4B.
- **Volume mesh convergence based on the finest surface mesh:** Based on the surface mesh consisting of 25,000 surface elements, mean aberration of measured local WSS values of the coarse meshes was 1.40% (95%-CI 1.07–1.72) with a maximum aberration of 3.98%. Results are shown in Fig. 4B. A numeric summary of all results of grid convergence is given in Table 3.

**Table 3:** Numeric summary of results from grid convergence: The table shows the results for surface grid convergence, convergence of volume meshes based on the surface defined with 15,000 elements and convergence of volume meshes based on the surface defined with 25,000 elements

	Mean relative aberration of coarse mesh (%, 95%-CI)	Max. relative aberration (%)	Mean relative aberration of medium mesh (%, 95%-CI)	Max. relative aberration (%)	Mean relative aberration of fine mesh (%, 95%-CI)	Max. relative aberration (%)
Surface grid convergence	10.07% (6.00–14.14%)	25.32%	2.30% (1.51–3.09%)	9.65%	Reference	Reference
Volume mesh convergence based on the surface consisting of 15,000 elements	2.41% (1.76–3.07%)	7.53%	2.38% (1.68–3.07)	7.39%	2.61% (1.93–3.29%)	7.59%
Volume mesh convergence based on the surface consisting of 25,000 elements	1.40% (1.07–1.72%)	3.98%			Reference	Reference

### Geometric mesh quality

Mean ICEM Quality of the analyzed meshes was in average 0.84 (95%-CI 0.83–0.85). Minimum ICEM Quality was in average 0.04 (95%-CI 0.03–0.05), maximum ICEM Quality 1.0 (95%-CI 0.999–1.00), It should be kept in mind that these minimum and maximum values might not sufficiently represent the quality of the whole mesh unlike the mean ICEM Quality.

### Time requirements

**Fig. 5:**
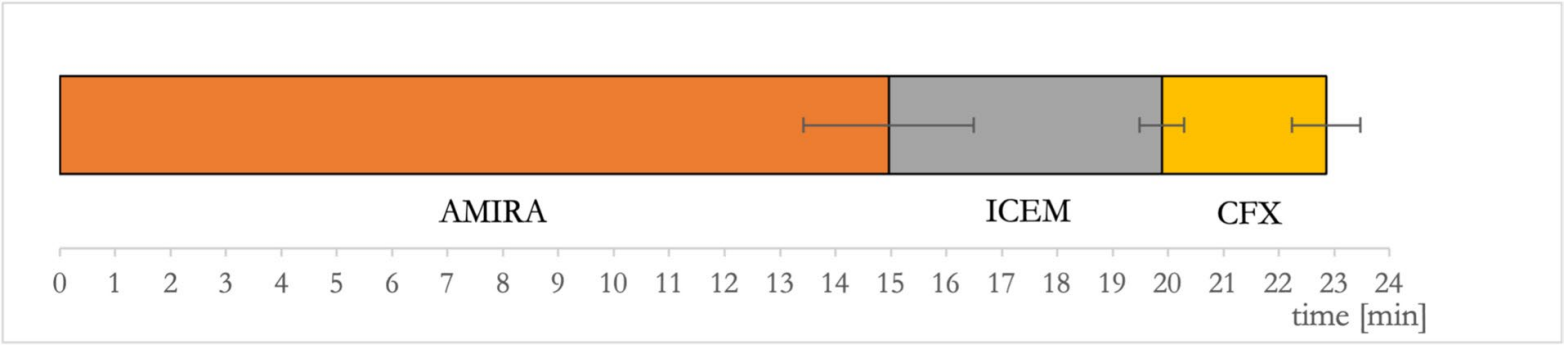
Results from time tests for the time needed for CFD simulations from DICOM images to postprocessed colormaps of CFD parameters in 5 M1 bifurcation aneurysms that were processed de novo: The mean time needed for calculations is shown in minutes, subdivided into the specific parts of the workflow (AMIRA, ICEM and CFX). Error bars indicate 95%-CI’s

 In average, time from original 3DRA-DICOM datasets to postprocessed colormaps took 22 min 51.4 s (95%-CI 20 min 51.562 s – 24 min 51.238 s). Most time consuming steps were cropping (5 min 43.6 s, 95%-CI 4 min 28.900 s – 6 min 58.300 s, manual) and smoothing (4 min 10.4 s, 95%-CI 3 min 46.118 s – 4 min 34.682 s, automatized). Results are visualized in Fig. 5. Additionally, processing times for selected specific steps of the workflow are given in Fig. 2A.

## Discussion

Hemodynamics are widely accepted to play a major role in aneurysm pathophysiology [20, 64, 66, 72, 75]. As vascular endothelial cells are able to sense mechanical forces and stimulate various pathways leading to different biological changes in the vessel wall, the most important factor for mechanotransduction is Wall Shear Stress (WSS) [3, 4, 16, 21, 34, 90]. WSS is promoting changes on biochemical [16], inflammatory [27, 64, 75, 80] and histological base [49, 51] and has been proved as one of the most important factors in aneurysm genesis, growth and rupture [20, 50, 51, 64, 66, 72, 75]. When balance between flow and pathobiology of the wall is disturbed, aneurysms become unstable resulting in enlargement or rupture [50]. Previous studies could show that areas of low WSS are related to thin and vulnerable wall regions prone to rupture [18, 41, 44, 70] as well as high pressure [71].

The aim of our study was to make clinicians benefit from hemodynamic information in the daily routine. Therefore, we developed a fast and reliable semi-automated workflow for aneurysm preprocessing, usable even in acute cases. The ease of handling makes the workflow suitable for clinical routine. Direct 3D-thresholding, 3D–cropping at the reconstructed geometry and direct visual comparison of 3D-models with raw data and overlays make the workflow intuitive and easy. No programming experience is needed which opens the toolchain to computationally unexperienced users. As correlations between low WSS and thin walled regions could be found consistently in steady state as well as pulsatile simulations [41], the use of steady state simulations seems feasible for clinical routine. For calculation of additional hemodynamic parameters like OSI, Aneurysm Formation Index (AFI) and Gradient Oscillatory Number (GON), transient simulations might be used, even if these were not assessed in this study.

Standardized parameter ranges were defined based on validations at 24 aneurysms to gain a good balance between rapidness and robustness. Therefore, the limitations of our study are primarily derived from the required balance between adequate CFD complexity, time requirements and usability. Further limitations of our study are, besides small sample sizes, the assumption of rigid walls and the use of only medium inlet lengths and mesh sizes. This allows rapid but robust results regarding unstable areas for clinical use even in urgent cases. Nevertheless, simulations using finer meshes, longer inlet- and outlet lengths as well as pulsatile flow might evoke more detailed results. Usage of medium meshes as defined for the grid convergence studies might be considered sufficient for many clinical cases, but eventually depending on the individual geometry of the parent vessels and also the geometry and size of the aneurysm. Validations with ultra-fine meshes and high-performance simulations, which might represent the hemodynamic ground-truth better than the finest meshes of our analysis were not part of this study. In a previous review, Berg et al. recently recommended an element size of 0.1 mm or less for adequate representation of the geometry [7]. As WSS is dependent on velocity gradients near the wall [63], regarding grid convergence, WSS is not solely dependent on the density of the surface mesh, but also the resolution near the vessel wall. In our study, prism layers were used providing a good compromise between local resolution near the wall and processing time, which were not altered in grid convergence studies, but might also bear an influence. WSS was chosen as the parameter of interest for grid convergence to estimate the concrete influence of grid density on this parameter as WSS is the major parameter of interest and best validated regarding the identification of thin-walled areas in literature. Results from meshes defined with 25,000 surface elements and a medium seed size of 0.25 mm were not analyzed within this part of the study as small changes in seed size used for volume meshing didn’t lead to relevant changes in element numbers any longer due to the two-step approach for the calculation of volume meshes (calculation of volume meshes based on surface meshes under protection of the surface elements). As maximum relative aberrations were mainly found at dome regions with markedly low WSS-values, absolute errors were mostly small, even if the concrete clinical influence of these errors is not fully clear yet.

Reconstruction of the vascular geometry was performed exclusively based on 3DRA’s within this study to guarantee adequate geometric representation, even if in urgent cases, the usage of CTA’s might better fulfill the clinical needs. Nevertheless, a previous review also recommended the usage of 3DRAs to ensure adequate reconstruction results for the subsequent CFD simulations [7]. As various smoothing processes provoke a shrinkage of 3D surfaces, a second threshold was applied after smoothing operations to compensate for even moderate shrinking artifacts. Adequate volumetric representation of the geometry was ensured by definition of the thresholds in direct overlay with the 3DRA data and final checks of reconstruction results regularly in each case. For definition of outlet pressures, flow distribution or coupling the simulation to a 1D-model of the vascular tree [57, 58] might provide better options regarding adequate CFD results than a zero-pressure condition [7]. Processing times were assessed for one operator and for 5 cases only in this study. As time requirements are dependent on a variety of aspects like computational training, the individual speed and learning curve of the operator, imaging quality, geometric complexity of the case etc., the presented data does not conclusively address the time requirements for all possible scenarios. Nevertheless, despite the small sample size and the additional influencing factors mentioned, these data are thought to provide a gross estimation of processing times. Between the investigated cases, no profound absolute differences in time were found despite different aneurysm sizes and different geometrical complexity. Lastly, no comparisons of final results were performed to existing software solutions previously proposed for aneurysm CFD like AView or Vmtk [2, 84] or other CFD tools like OpenFOAM [92]. As individual requirements relevantly differ between individual operators, these workflows might also provide a sound approach to perform CFD simulations in the clinical context depending on the individual requirements. The advantages of the workflow developed within this study might be seen in the ease of handling with interactions mainly directly at the 3D-geometry, no need of programming skills and the definition of standard parameter ranges used for simulations.

A variety of methodological influences have been previously discussed with respect to CFD like.

- the impact of segmentation and different imaging modalities (for example [33, 56] amongst other studies),
- intrinsic methodological aspects like solver numerics, mesh- and time step resolution and solution strategies ([45, 76] amongst others),
- the usage of different rheological models and inflow conditions ([11, 24, 78, 82, 86] amongst others) as well as
- the validation of CFD results with different modalities ([30, 42, 87] amongst others).

Because of this variety of possible influences and parameters to consider, different challenges compared CFD results of different authors [5, 36, 68, 77]. In one study, constant solutions for pressure could be found, although some differences with respect to flow patterns and pressure derivatives existed, even if the practical significance of these differences is not clear. The influence of inlet boundary conditions has clearly been underlined by the authors [68]. Another study also found likewise solutions of a given CFD problem amongst different workgroups in approximately 80% of the cases, even if a variety of workflows had been used [5]. A ruptured vs. a non-ruptured case could have been correctly identified by the majority of the groups in another challenge. Interestingly, in a comparative group of neurosurgeons analyzing only geometric 3D images lacking hemodynamic information using a double-blinded questionnaire, none of the participants could correctly identify the site of rupture [36], which additionally underlines the need of hemodynamic simulations. Even if still not all factors underlying aneurysmal hemodynamics are fully clear yet and also previous literature partially reported opposing results [61, 83], the potential benefit of the method for clinicians has previously been discussed repeatedly [6, 14, 17, 35].

Multiple reasons prevent CFD to come into clinics, even if a previous study found acceptable CFD results in a CFD workshop with mainly clinical participants [65]. The authors underline the clinical need of hemodynamic simulations, which found a clear majority in a survey among the participants. Probably, the most important obstacles are i) the complicated handling of existing CFD workflows, needed programming experience and the main usage by experts in science and labs, ii) the lack of knowledge and experience to define optimum parameters for each geometry to gain stable results and iii) the amount of time needed for simulations. In general, the main benefits of this workflow might therefore be seen in

1. Direct manipulation straight on the 3D-view in most of user-dependent steps, especially for reconstruction of the vascular geometry not requiring programming skills,
2. Avoidance of excessive processing time due to compromises between CFD complexity and time requirements in the clinical setting,
3. Analysis of standardized parameter ranges used for the specific settings of CFD simulations,

which could help to make clinicians benefit from the availability of hemodynamic data during surgery or interventional procedures. The aim of our study was not to fully address the complexity of methodological pitfalls of CFD simulations, but to develop a simple and intuitive workflow with a special respect to clinical feasibility and reasonable applicability. Therefore, additional methodological aspects might exist, that were not finally addressed within this work.

In conclusion, CFD simulations using the presented settings are thought to provoke a good overview for clinical application with acceptable processing times, which are mainly made possible due to a relatively low spatial resolution compared to other CFD studies and the usage of steady state simulations, but might not be detailed enough for basic science. Nevertheless, the workflow can also be used for these questions with different parameters resulting in longer processing times. Though, general risk assessment or the decision whether to operate or not based on CFD will probably take some more time, the described workflow enables the operating neurosurgeon or neurointerventionalist to gain a fast overview over unstable areas of the aneurysm and thin-walled regions with possible point of rupture. This might be helpful to adopt the intraoperative dissection strategy individually to the specific aneurysm and could provide an additional valuable puzzle piece amongst other pre- and intraoperative imaging modalities.

## Data Availability

Data supporting the findings of this study are available from the corresponding author upon reasonable request.

## Abbreviations

AFI: Aneurysm formation index
CTA: Computed tomography angiography
CFD: Computational fluid dynamics
CGI: Grid convergence index
GON: Gradient oscillatory number
GPA: Granulomatosis with polyangiitis
MRI: Magnetic resonance imaging
MRA: Magnetic resonance angiography
OSI: Oscillatory shear index
ROI: Region of interest
SAH: Subarachnoid hemorrhage
TCD: Transcranial doppler ultrasound
WSS: Wall shear stress
WSSG: Wall shear gradient
3DRA: Three-dimensional rotational angiogram
